# Report of the Committee on Communication from the American Medical Association in Relation to Licenses

**Published:** 1870-08

**Authors:** N. S. Davis, David Prince


*Report of the Committee on Communication from the American Medical Association in relation to Licenses:*

Your Committee, to whom was referred the following communication from the American Medical Association, respectfully report as follows:

The communication is as follows:

*Whereas,* The history of medical legislation in the various States of this Union, clearly shows that no reliance can be placed on either the uniformity or the permanency of any laws relating to the practice of medicine; and

*Whereas,* The results of all the efforts made during the last twenty-five years, to elevate the standard of medical education, through concert of action among the numerous medical colleges of this country, have proved with equal clearness that such concert of action, in an efficient manner, is unattainable; therefore, be it

*Resolved,* That whatever is done to establish and maintain a just and fair standard of medical education throughout our whole country, must be done by the profession itself, through its own voluntary organizations, in the same manner that it now establishes and enforces its code of ethics. The profession is as competent to declare, through its representatives in the National, State, and local Societies, what shall be the standard of attainments for those to be recognized and admitted into its ranks, and to establish the boards or agencies by which compliance with such standard shall be ascertained, as it is to declare what shall be the ethical rules governing the conduct of those already admitted.

*Resolved*, That this Association earnestly request each State Medical Society to appoint annually one or more Boards of Examiners, composed of five thoroughly competent members, whose duty it shall be to meet, at suitable times and places, for the examination of all persons, whether graduates of colleges or not, who propose to enter upon the practice of medicine in their respective States, except such as have been previously examined and licensed by a similar board, in some other State.

*Resolved*, That each State Medical Society be requested to make such regulations concerning the pay of the Boards of Examiners, and the fee to be charged for a license to practice, that the former shall in no case depend on the amount received from the latter.

*Resolved*, That each State Medical Society be requested to require its Examining Board, or Boards, to exact of every applicant for examination, adequate proof that he has a proper general education; is twenty-one years of age, and has pursued the study of medicine three full years, one-half of which time shall have been in some regularly-organized medical college, whose curriculum embraces adequate facilities for didactic, demonstrative, and hospital clinical instruction.

*Resolved*, That each State Medical Society be requested to act on the foregoing propositions, at the next regular annual meeting after the reception of copies of the same, and if approved and adopted by the State Medical Societies of two-thirds of the States, this Association shall deny representatives from all organizations who longer refuse to comply with the same, and shall recommend the State Societies to do the same, and all persons who, after that date, seek to enter upon the practice of medicine without, first receiving a license from the State Board of Examiners, shall be treated ethically as irregular practitioners.

*Resolved*, That in adopting the foregoing resolutions, by which it is proposed to treat the Medical College diploma the same as the diploma of any literary college, this Association is actuated by no desire to injure the Medical Schools of our country. On the contrary, by the adoption of the fourth resolution at the same time that the value of the mere college diploma is practically nullified, it is the desire, and confident expectation, that those institutions will be greatly benefited; because they will be forced to rival each other in the extent and efficiency of their courses of instruction, instead of the number of diplomas which they can annually distribute.

The subject thus presented is one of great importance, and should receive a very careful consideration on the part of the profession. The communication contains three distinct topics, capable of separate consideration.

1st. The facts asserted in the preamble.

2d. The right and the duty of the profession to establish and maintain a just and fair standard of medical education for all such as are admitted into its ranks, and recognized as regular practitioners.

3d. The particular mode for establishing and enforcing such standard of education.

The facts asserted in the preamble are, that the history of State legislation on the one hand, and of medical college action on the other, have fully demonstrated such a degree of discordance and instability in the first, and such want of concert and faithfulness in the second, that neither can be relied upon, either to establish or enforce a just and proper standard of education for the profession. That these assertions of the preamble are true to the full extent, is too obvious to all who have examined the subject, to require any proof. To procure the passage of a law, substantially the same in relation to a standard of medical education, by thirty or more State legislatures, would be a work of great difficulty; and to keep such a law intact on the statute books of all the States for any length of time, against all the various combinations of medical and political quackery, would be practically impossible. If any doubt this, we would refer them to the history of medical education and institutions in the

United States, which they can find in a little volume published in 1851.

Every effort to procure concert of action on the part of the medical colleges has hitherto proved equally fruitless. For more than twenty years these institutions have been urged by the profession, speaking through the American Medical Association, to adopt and enforce a higher standard of requirements for those proposing to enter the profession. Four distinct efforts have been made to effect the desired concert of action, by conventions of delegates from medical colleges exclusively. The first was in Louisville, in 1859; the second in New Haven, in 1860; the third in Cincinnati, in 1867; and the fourth in Washington, in 1870. The two last-named conventions were attended by delegates from about half of the medical colleges in the United States; and in both, after full discussion, a very satisfactory plan of medical education was agreed upon, and strongly recommended for adoption.

But while thus agreeing as to what *ought* to be done, a large part of the delegates, especially from the Eastern schools, disclaimed having any authority to make the recommendation binding upon their respective colleges; and consequently it, like the numerous resolutions passed by the American Medical Association on the same subject, was of no practical effect, except as an expression of opinion. We think, therefore, it may be safely assumed that the assertions of the preamble are strictly correct. This being conceded, there are, logically, but two alternatives left:

One, to let the subject of medical education remain in *statu quo*, with no standard of preliminary qualifications on the part of students, no system or order in the pursuit of strictly medical studies, and no uniform or disinterested tribunal for determining the qualifications of those who are to be admitted into the profession.

The other is, to adopt and carry into practical effect the propositions in the first resolution in the communication under consideration; namely, that it is both the right and the duty of the profession to establish and maintain the standard of acquirements deemed necessary for those who are to be admitted and recognized as members of such profession. And to do this through the agency of its representatives in the State and National organizations.

The present educational condition of the medical profession in this country is peculiar, and without a parallel in any of the other professions. Absolutely without any practical standard of education for those who propose to study medicine; without any expressed or recognized standard of attainments in medicine to be exacted of candidates for admission; we present the spectacle of a great and important profession standing with folded arms, and receiving complacently whoever forty or fifty independent medical schools may see fit to commission with the title of M. D.; the faculties of such schools being at once the paid instructors and sole judges of the moral, intellectual, and professional qualifications of those they thus commission. No such relation exists between the profession and the colleges in any other country; and no parallel relation exists, either in the professions of law or divinity in our own country. The judges of the various courts are the tribunals before whom the applicants for admission to the bar must substantiate their qualifications; and the diocesans, presbyters, synods, and conferences of the various religious denominations determine the qualifications of those who are to be admitted into the clerical ranks.

The schools of law and of theology are established for purposes of instruction solely; and the same should be true of schools of medicine. If this were the case, or if the profession, through its own organizations, established its standard of requirements and its own tribunals or boards of censors for exacting compliance with such standard, it would speedily change the direction of the rivalry among the colleges and the influences controlling the students in choosing the colleges they would attend. While the mere college diploma remains, as at present, a full passport into the profession, a controlling question with a large part of students will be, not where can they get the most thorough qualifications in a given length of time, but where can they reach a diploma for the least expenditure of time and money; and in the same proportion will the schools rival each other, not in the extent and perfection of their courses of instruction, but in the ease and cheapness with which they can offer their diplomas, and yet preserve the form of respectability. An experience of more than twenty years in connection with medical college instruction, aided by an active intermingling with all interests in the profession, has fully satisfied us that the propositions submitted by the American Medical Association are founded on correct principles, and look to the adoption of a policy of great practical importance. And hence we hope they will meet with the cordial sanction of this Society.

There may be some, however, who, though admitting the correctness of the proposed policy, nevertheless regard it as incapable of practical application. Such claim that every one practices medicine who pleases, and that boards of censors appointed by State Medical Societies, having no legal authority, would be unheeded by a large part of those entering into the ranks of the profession.

It is *true*, that in our country, any one prescribes for the sick who pleases; but it does not follow that every one who prescribes is recognized as a member of the profession, consulted with, and made eligible to membership in our medical societies. On the contrary, certain rules of ethics, voluntarily adopted by our medical organizations, having no sanction of civil law, but distinctly indicating conditions of recognition for members of the profession, are very generally respected and obeyed. So if every State Medical Society would appoint proper boards of censors who, after a given date, should be ready to thoroughly examine every candidate for admission into the ranks of the profession, and the submission of every candidate to such examination and the procurement of a license was made a positive requirement for eligibility to membership in the local, State, or National Medical Secieties, and for recognition as a *regular practitioner*, it would soon come to be as universally complied with as any other well-established rule of the profession. There is an instinctive desire in the mind of every educated member of an enlightened profession to stand well with his brethren, and to be fully eligible to every privilege and immunity belonging to that profession. And this feeling, especially if aided by public sentiment, commands far more implicit obedience in our country than any statutory law.

There is one department of the education of our profession, the regulation of which so plainly belongs to the profession at large, that we call your special attention to it. We allude to the preliminary education necessary before commencing medical studies proper. It cannot be reasonably denied that a very large part of the evils in our profession depend on the imperfect general education of those who enter it. The desirableness and necessity of a fair degree of mental development and discipline, with a knowledge of the common branches of education, including the physical sciences, as a preparation for the study of medicine proper, is so obvious to every reflecting mind, that comments on the subject are superfluous. The honor and usefulness of the profession imperatively demand the universal adoption of a fair standard of preliminary education, and its rigid enforcement, as a condition of admission both into the office of any physician and as a matriculate in a medical college.

In view of the foregoing observations, we would close this report by offering for your consideration the two following resolutions:

1st. *Resolved*, That this Society cordially approves the propositions contained in the resolutions received from the American Medical Association, and quoted in the commencement of this report.

2d. *Resolved*, That this Society approves of the standard of preliminary education agreed upon by the Convention of delegates from medical colleges, in Cincinnati, in 1867, and re-approved by the Convention in Washington, in May, 1870, except so much as relates to Greek and Latin; and that it is the duty of every State and local medical society to appoint annually a board of censors, whose duty it shall be to examine all persons who propose to enter upon the study of medicine in their respective districts, and to give a written certificate of qualifications. And further, that where such *boards* exist, it shall be considered a violation of the ethics of the profession for any practitioner to receive a student into his office before he has procured a satisfactory certificate from such board.

Respectfully submitted.

N. S. DAVIS,
DAVID PRINCE.

